# *RUNX1* haploinsufficiency results in granulocyte colony-stimulating factor hypersensitivity

**DOI:** 10.1038/bcj.2015.105

**Published:** 2016-01-08

**Authors:** D W L Chin, M Sakurai, G S S Nah, L Du, B Jacob, T Yokomizo, T Matsumura, T Suda, G Huang, X-Y Fu, Y Ito, H Nakajima, M Osato

**Affiliations:** 1 Cancer Science Institute of Singapore, National University of Singapore, Singapore; 2 Division of Hematology, Department of Internal Medicine, Keio University School of Medicine, Tokyo, Japan; 3 Institute of Molecular and Cell Biology, A*STAR, Singapore; 4 International Research Center for Medical Sciences, Kumamoto University, Kumamato, Japan; 5 Department of Paediatrics, Cincinnati Children's Hospital, Cincinnati, OH, USA; 6 Department of Biochemistry, Yong Loo Lin School of Medicine, National University of Singapore, Singapore; 7 Institute of Bioengineering and Nanotechnology, A*STAR, Singapore; 8 Department of Paediatrics, National University of Singapore, Singapore

## Abstract

*RUNX1/AML1* is among the most commonly mutated genes in human leukemia. Haploinsufficiency of *RUNX1* causes familial platelet disorder with predisposition to myeloid malignancies (FPD/MM). However, the molecular mechanism of FPD/MM remains unknown. Here we show that murine *Runx1*^+/−^ hematopoietic cells are hypersensitive to granulocyte colony-stimulating factor (G-CSF), leading to enhanced expansion and mobilization of stem/progenitor cells and myeloid differentiation block. Upon G-CSF stimulation, *Runx1*^+/−^ cells exhibited a more pronounced phosphorylation of STAT3 as compared with *Runx1*^+/+^ cells, which may be due to reduced expression of Pias3, a key negative regulator of STAT3 signaling, and reduced physical sequestration of STAT3 by RUNX1. Most importantly, blood cells from a FPD patient with RUNX1 mutation exhibited similar G-CSF hypersensitivity. Taken together, *Runx1* haploinsufficiency appears to predispose FPD patients to MM by expanding the pool of stem/progenitor cells and blocking myeloid differentiation in response to G-CSF.

## Introduction

*RUNX1* is one of the most frequently mutated genes in human leukemia. Approximately 30% of acute leukemias carry *RUNX1* genetic alterations such as chromosomal translocation, point mutations and deletions.^1, 2^ A germline mutation of *RUNX1* is causally linked to familial platelet disorder with predisposition to myeloid malignancies (FPD/MM), a rare autosomal dominant disorder characterized by modest thrombocytopenia and defective platelet function, with 35% lifetime risk of developing leukemia.^3, 4, 5, 6^ To date, ~30 families have been reported.^7^ Most of the *RUNX1* mutations identified in these patients concentrate within the Runt domain and disrupt the DNA binding and β heterodimerization capabilities.^1^ In some cases, mutations are also found in the carboxyl terminus, abrogating the transactivation domain and resulting in formation of dominant negative forms of RUNX1.^4^

*Runx1* is well established as a master regulator of hematopoiesis. *Runx1*^*−/−*^ murine embryos die at embryonic day 12.5 due to hemorrhage in the central nervous system and inability to generate hematopoietic stem cells (HSCs).^8, 9^ Inactivation of *Runx1* at the adult stage using *Runx1* conditional knockout mice results in expansion and subsequent exhaustion of hematopoietic stem and progenitor cells (HSPCs).^10, 11^ *Runx1* deficiency is insufficient for leukemogenesis and requires the accumulation of additional mutations for transformation.^11^ *Runx1* haploinsufficiency is also insufficient for leukemogenesis, although mild phenotypes such as reduced platelet counts and elevated hematopoietic progenitor counts were observed in *Runx1*^+/−^ mice, which are genetically identical to FPD patients.^12, 13^ It remains unclear how *RUNX1* haploinsufficiency promotes leukemogenesis in FPD patients.

HSC behaviors such as self-renewal, proliferation and mobilization are tightly orchestrated by cell intrinsic and extrinsic factors, the latter of which includes secreted factors and cell–cell interactions within the bone marrow (BM) niche.^14, 15, 16^ Granulocyte colony-stimulating factor (G-CSF) is a potent cytokine that induces HSPC proliferation, mobilization and promotion of granulopoiesis.^17, 18^ Many infections trigger stressed granulopoiesis through the production of G-CSF to augment granulocyte differentiation. G-CSF is clinically used to mobilize and collect HSCs for peripheral blood stem cell transplantation.^19^ G-CSF also alleviates severe neutropenia in severe congenital neutropenia patients.^20^ Recently, there has been growing evidence that suggests an intimate link between RUNX1 and G-CSF signaling. Mutations in *RUNX1* and G-CSF receptor (*CSF3R*) genes were concurrently found in severe congenital neutropenia patients who developed acute myeloid leukemia.^21^ Treatment with G-CSF and the loss of RUNX1 enhance the detachment of long-term HSCs from the niche and increase the frequency of short-term HSCs.^16^ We recently reported that deficiency of *Runx3*, a closely related member of RUNX family, also results in G-CSF hypersensitivity.^22^

To better understand how *Runx1* haploinsufficiency contributes to leukemogenesis, the steady-state hematopoiesis and cytokine responses of *Runx1*^+/−^ mice were carefully examined. *Runx1*^+/−^ mice exhibited G-CSF hypersensitivity as evidenced by HSPC expansion and mobilization to the periphery, as well as myeloid differentiation block. Importantly, FPD patient-derived peripheral blood mononuclear cells harboring a *RUNX1* point mutation demonstrated similar G-CSF hypersensitivity when compared with healthy donor cells. These results suggest that Runx1 haploinsufficiency can increase the pool of immature progenitor cells, thereby increasing the probability of acquiring cooperative mutations for leukemic transformation.

## Materials and Methods

### Mice and G-CSF stimulation

*Runx1*^+/−^ mice were generated by Okada *et al.*^23^ and maintained in the C57BL/6 background. Experiments were carried out using 6 to 8-week-old littermates. For *in vivo* G-CSF administration, mice were subcutaneously injected with 250 μg/kg/day murine G-CSF or phosphate-buffered saline daily for three consecutive days. Peripheral blood (PB) was obtained via retro-orbital bleeding. Mice were killed at 24 or 72 h after the final injection. BM cells were harvested by flushing femurs and tibias in ice-cold phosphate-buffered saline and incubated with red blood cell lysis buffer. All experimental procedures were approved by Institutional Animal Care and Use Committee (IACUC).

### FPD patient

PB samples from subjects were collected after obtaining written informed consent. The study was conducted with approval from the internal review board of Keio University School of Medicine, Tokyo, Japan and conformed to the principles outlined in the Declaration of Helsinki for use of human tissue or subjects.

### Colony-forming unit-culture (CFU-C) assay

Fifty or ten thousand murine whole-BM cells, 100-sorted HSPCs/ myeloid progenitors or 20 μl of PB were seeded into 35 mm dishes in Methocult (M3231, StemCell Tec., Vancouver, BC, Canada) supplemented with 10 or 100 ng/ml murine G-CSF, 10 ng/ml granulocyte-macrophage CSF, 10 ng/ml interleukin-3 (IL-3), 500 ng/ml interleukin-6 (IL-6) and 100 ng/ml stem cell factor. All cytokines were purchased from Peprotech (Rocky Hill, NJ, USA). Cell cultures were incubated at 37 ^o^C, 5% CO_2_ and colonies number were scored after 10 days. CFU-C assay for FPD patient was performed as previously described.^7^

### Flow cytometry

Flow cytometric analysis and sorting were performed using LSR II Flow cytometer and FACSAria instrument (BD Biosciences, Franklin Lakes, NJ, USA), respectively. Monoclonal antibodies were purchased from BD Pharmingen (Franklin Lakes, NJ, USA) or eBioscience (San Diego, CA, USA). Antibodies used are listed in Supplementary Table 1.

### Luciferase assay

The luciferase reporter plasmid pGL3-CXCR4-luc,^24^ pGL3-PIAS3-luc and effector RUNX1 plasmids were transfected into HL-60 cells using FuGENE 6 (Promega, Madison, WI, USA). Equal amounts of DNA were transfected by supplementing appropriate amounts of the backbone pEF plasmid. Forty-eight hours after transfection, cells were lysed with 1 × passive lysis buffer, and the luciferase assay was performed using the Dual-Luciferase Reporter Assay Kit (Promega).

### RNA extraction, complementary DNA conversion and real-time PCR

Total RNA was extracted using Nucleospin RNA XS kit (Macherey-Nagel, Dueren, Germany). Equal amounts of RNA were reverse transcribed into complementary DNA with Superscript III reverse transcriptase (Life Technologies, Carlsbad, CA, USA). Power SYBR master mix or Taqman gene expression master mix (Life Technologies) were used for real-time PCR analysis. The primers or taqman probes used are listed in Supplementary Table 2.

### Statistical analysis

Differences between samples were evaluated by two-tailed Student's *t*-test. All values are expressed as mean±s.d. unless otherwise stated. *P*-values<0.05 were considered statistically significant.

## Results

### *Runx1*^ +/−^ BM cells exhibit cytokine sensitivity *in vitro*

To investigate the impact of *RUNX1* haploinsufficiency on hematopoiesis and leukemogenesis in FPD/MM patients, we used mice that are heterozygous for the *Runx1* null mutation (*Runx1*^+/−^). Complete blood count analysis of 6–8-week-old *Runx1*^+/−^ mice showed normal frequency of white blood cells and hemoglobin, but a significant reduction in platelet counts (Supplementary Figure 1A). *Runx1*^+/−^ mice did not exhibit major differences in the percentages of Mac-1^+^Gr-1^+^ granulocytes, B220^+^CD19^+^ B cells and CD3^+^ T cells within the BM, compared with *Runx1*^+/+^ mice (Supplementary Figure 1B). Despite *Runx1*^+/−^ mice having reduced platelet counts, CD41^+^CD61^+^ megakaryocytes were modestly increased in the BM (Supplementary Figure 1B), probably due to blockage in megakaryocyte differentiation. The spleen of *Runx1*^+/−^ mice showed increased granulocytes but decreased T-cell frequencies (Supplementary Figure 1C). Further examination of the HSPCs and myeloid progenitor compartments in the BM showed that the cKit^+^Sca-1^+^Lin^*−*^ (KSL) and cKit^+^Sca-1^-^Lin^*−*^ (KL) fractions were comparable between *Runx1*^+/+^ and *Runx1*^+/−^ mice (Supplementary Figure 1D). However, higher numbers of KSL and KL cells were consistently detected in the spleen of *Runx1*^+/−^ mice (Supplementary Figure 1E). As compared with previous reports, which described HSPC expansion, lymphopenia and thrombocytopenia in *Runx1* conditional knockout mice,^10, 11^ *Runx1*^+/−^ mice display weaker hematopoietic phenotypes. Importantly, the reduction in platelet count of *Runx1*^+/−^ mice mimics the mild thrombocytopenia observed in FPD patients.

**Figure 1:**
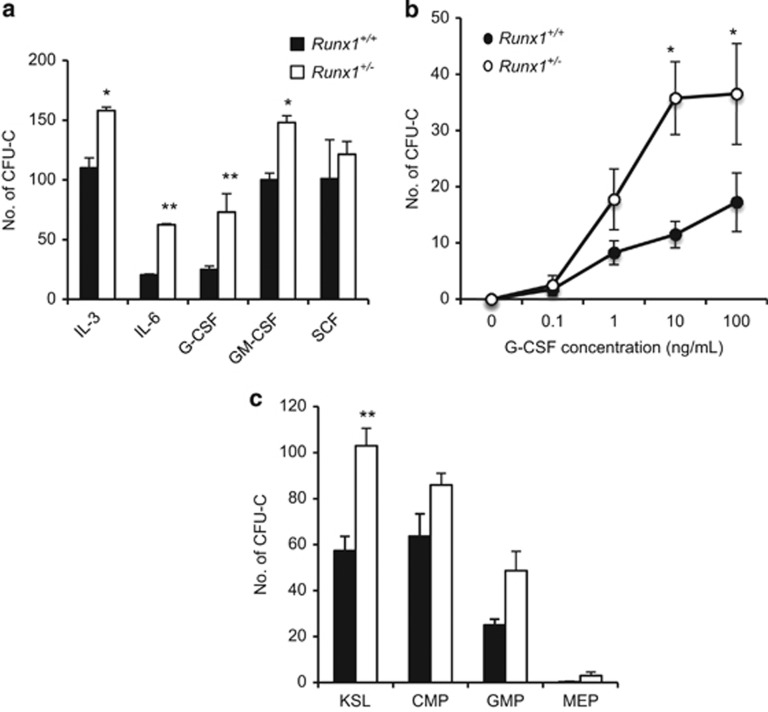
*Runx1*^*+/*−^ BM cells show G-CSF hypersensitivity *in vitro*. (**a**) Colony-forming potential of 50 000 whole-BM cells in the presence of indicated cytokine: 10 ng/ml IL-3, 500 ng/ml IL-6, 10 ng/ml murine G-CSF, 10 ng/ml granulocyte-macrophage CSF (GM-CSF) and 100 ng/ml stem cell factor (SCF). (*n*= 3 mice/genotype). (**b**) Colony-forming potential of 10 000 whole-BM cells in the presence of increasing concentration of G-CSF. (*n*= 3 mice/genotype). (**c**) Colony-forming of purified progenitor compartments in the presence of 100 ng/ml G-CSF. All data represent mean±s.d. (*n*= 3 mice/genotype). Three independent experiments were performed. CMP, common myeloid progenitors (CD34+FcγRII^-^KL); GMP, granulocyte-macrophage progenitors (CD34+FcγRII^+^KL); MEP, megakaryocyte erythrocyte progenitors (CD34-FcγRII^-^KL). Asterisks represent significant differences (*P<0.05, **P<0.01 and two-tailed Student's *t*-test).

We next investigated cytokine sensitivity of *Runx1*^+/−^ cells. Whole-BM cells from *Runx1*^+/+^ and *Runx1*^+/−^ mice were cultured in methylcellulose supplemented with a single factor chosen from a panel of cytokines: IL-3, IL-6, G-CSF, granulocyte-macrophage CSF and stem cell factor. *Runx1*^+/−^ BM cells formed twice the number of colonies of wild-type cells after stimulation with IL-6 and G-CSF, and to a lesser extent in the presence of IL-3 and granulocyte-macrophage CSF (Figure 1a). These observations indicate that *Runx1*^+/−^ status enhances multiple cytokine responses. G-CSF and IL-6 share a common downstream signaling molecule, STAT3. Owing to the known association between Runx1 and G-CSF in MM stated earlier, we chose to focus our experiments on G-CSF. *In vitro* stimulation of whole-BM cells with increasing dosage of G-CSF revealed that *Runx1*^+/−^ BM cells formed significantly more colonies at concentrations of 10 and 100 ng/ml G-CSF (Figure 1b). To further identify the exact cellular population that is hyper-responsive to G-CSF, KSL and the various myeloid progenitors were sorted and treated with G-CSF *in vitro*. The colony-forming capacity of the KSL compartment of *Runx1*^+/−^ mice showed the greatest difference when compared with littermate control, though this trend of G-CSF hypersensitivity was also observed in other myeloid progenitor compartments (Figure 1c). The results from the *in vitro* colony-forming assays demonstrate that *Runx1* haploinsufficiency promotes hypersensitivity to G-CSF of HSPCs.

### *Runx1*^ +/−^ hematopoietic cells are hypersensitive to G-CSF *in vivo*

**Figure 2:**
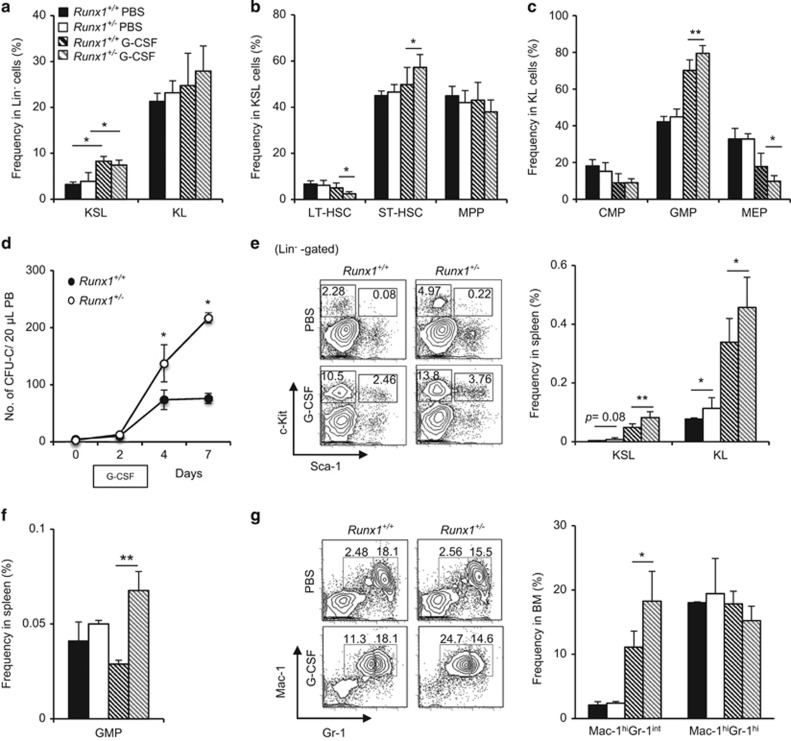
*Runx1*^+/−^ mice exhibit acute G-CSF hypersensitivity *in vivo.* (**a**–**c**) Graphical representation of flow cytometric analysis of the HSPCs and myeloid progenitor compartments (**a**), individual HSPC compartments (**b**) and myeloid progenitors (**c**) in the BM 24 h after *in vivo* G-CSF stimulation (250 μg/kg/day for 3 days). Percentages shown are frequencies of viable lineage^*−*^ population. Data from at least two independent experiments are shown. *(Runx1*^+/+^mice phosphate-buffered saline (PBS) control, *n*=4; *Runx1*^+/−^ mice PBS control, *n*=4; *Runx1*^+/+^mice G-CSF-treated, *n*= 6; *Runx1*^+/+^mice G-CSF-treated, *n*=6). (**d**) G-CSF mobilization assay of cells from *Runx1*^+/−^ mice and *Runx1*^+/+^ mice. Time course of colony numbers in 20 μl PB after *in vivo* G-CSF stimulation (250 μg/kg/day for 3 days) is shown. (*n*=2/genotype). Two independent experiments were performed. (**e**) Representative plots and graphical representation (left and right panel respectively) of flow cytometric analysis of the HSPC compartments in the spleen 24 h after *in vivo* G-CSF stimulation (250 μg/kg/day for 3 days). Percentages shown are frequencies of viable lineage^*−*^ population. *(Runx1*^+/+^ mice PBS control, *n*=4; *Runx1*^+/−^ mice PBS control, *n*= 4; *Runx1*^+/+^mice G-CSF-treated, *n*= 6; *Runx1*^+/+^ mice G-CSF-treated, *n*=6). (**f**) Representative graphical representation of flow cytometric analysis of GMP frequency in the spleen 24 h after *in vivo* G-CSF stimulation (250 μg/kg/day for 3 days). All data represent mean±s.d. *(Runx1*^+/+^mice PBS control, *n*=3; *Runx1*^+/−^ mice PBS control, *n*=3; *Runx1*^+/+^mice G-CSF-treated, *n*=3; *Runx1*^+/+^mice G-CSF-treated, *n*= 3). (**g**) Representative plots and graphical representation (left and right panel respectively) of flow cytometric analysis of the myeloid cells in the BM 24 shown *(Runx1*^+/+^mice PBS control, *n*=2; *Runx1*^+/−^ mice PBS control, *n*=2; *Runx1*^+/+^ mice G-CSF-treated, *n*= 4; *Runx1*^+/+^mice G-CSF-treated, *n*=4). CMP, common myeloid progenitors (CD34+FcγRII^-^KL); GMP, granulocyte-macrophage progenitors (CD34+FcγRII^+^KL); MEP, megakaryocyte erythrocyte progenitors (CD34-FcγRII^-^KL). Asterisks represent significant differences (*P<0.05, **P<0.01 and two-tailed Student's *t*-test).

To determine if *Runx1*^+/−^ mice also exhibit G-CSF hypersensitivity *in vivo*, *Runx1*^+/+^ and *Runx1*^+/−^ mice were treated with 250 μg/Kg/day G-CSF for three consecutive days and analyzed 24 h after the final injection. G-CSF treatment resulted in an increase in the percentage of KSL cells within the Lin^*−*^ compartment in both *Runx1*^+/−^ mice and its littermate controls (Figure 2a, Supplementary Figure 2A). Within the KSL compartment, G-CSF treatment significantly reduced the frequency of CD34^-^Flt3^-^KSL long-term HSCs of *Runx1*^+/−^ mice as compared with treated wild-type control (Figure 2b, Supplementary Figure 2B). This observation was accompanied by a minor increase in the frequency of CD34^+^Flt3^-^KSL short-term HSCs (Figure 2b, Supplementary Figure 2B). Although G-CSF did not significantly alter BM myeloid progenitor frequencies (KL fraction in Figure 2a), the frequency of CD34^+^FcγR^hi^KSL granulocyte-macrophage progenitors (GMPs) was increased, whereas that of CD34^-^FcγR^lo^KSL megakaryocyte erythrocyte progenitors was reduced in *Runx1*^+/−^ mice as compared with littermate control (Figure 2c, Supplementary Figure 2C).

As G-CSF is a potent agent for mobilization of HSPCs, we compared the HSPC mobilization between *Runx1*^+/+^and *Runx1*^+/−^ mice after G-CSF administration. When assessed at 24 and 72 h after the final G-CSF injection (days 4 and 7, respectively), PB cells from *Runx1*^+/−^ mice formed more colonies than *Runx1*^+/+^ mice in methylcellulose culture (Figure 2d), reflecting an elevated number of migrating HSPCs in *Runx1*^+/−^ mice. In agreement with the PB CFU-C assay, significantly higher frequencies of KSL and KL cells were also detected in the spleen of G-CSF-treated *Runx1*^+/−^ mice compared with those of *Runx1*^+/+^ mice (Figure 2e). Moreover, G-CSF treatment also resulted in a significant increase in splenic GMP frequency in *Runx1*^+/−^ mice as compared with treated control mice (Figure 2f). These results clearly demonstrate that *Runx1*^+/−^ HSPCs were more responsive to G-CSF and readily mobilized out of the BM.

To determine the effect of G-CSF on the differentiation of myeloid cells, we assessed the frequencies of Mac-1^hi^Gr-1^hi^ and Mac-1^hi^Gr-1^int^ granulocytes in the BM. G-CSF treatment of *Runx1*^+/+^ mice showed a fourfold increase in the percentage of Mac-1^hi^Gr-1^int^ granulocytes in the BM as compared with phosphate-buffered saline-treated control mice (Figure 2g). Interestingly, G-CSF treatment of *Runx1*^+/−^ mice resulted in eightfold increase in the Mac-1^hi^Gr-1^int^ fraction, accompanied by modest decrease in Mac-1^hi^Gr-1^hi^ granulocytes (Figure 2g). These results suggest that *Runx1* haploinsufficiency impedes G-CSF-induced granulopoiesis, which leads to accumulation of both GMP and Mac-1^hi^Gr-1^int^ granulocytes in the BM.

To further evaluate whether *Runx1*^+/−^ mice also show prolonged response to G-CSF, we performed similar analysis 72 h after the final G-CSF administration. The frequencies of the various HSPC and myeloid progenitor fractions in the BM were also comparable (Supplementary Figures 3A–C). However, the frequencies of splenic KSL and KL cells in *Runx1*^+/−^ mice remained significantly higher than those in *Runx1*^+/+^mice and steady-state *Runx1*^+/−^ mice (Supplementary Figure 3D). The increase in GMPs in the spleen of *Runx1*^+/−^ mice was also observed like steady-state *Runx1*^+/−^ mice (Supplementary Figure 3E), whereas the percentages of Mac-1^hi^Gr-1^hi^ and Mac-1^hi^Gr-1^int^ granulocytes in the BM were almost comparable between *Runx1*^+/−^ and *Runx1*^+/+^mice (Supplementary Figure 3F). These results suggest that *Runx1* haploinsufficiency causes G-CSF hypersensitivity, resulting in prolonged mobilization of HSPCs.

### RUNX1 negatively regulates STAT3 activation

**Figure 3:**
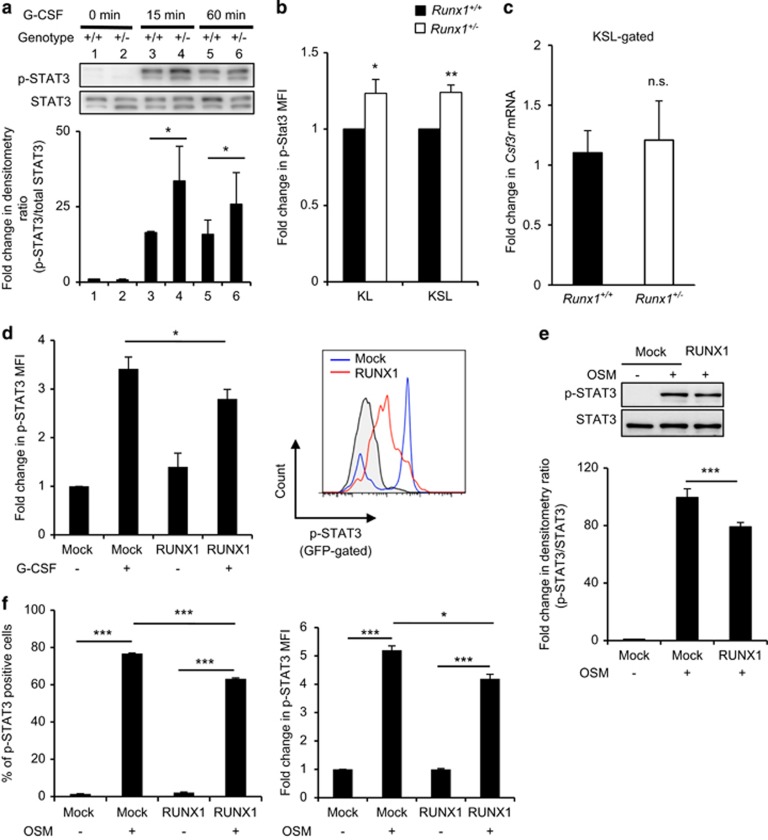
RUNX1 attenuates STAT3 phosphorylation after cytokine treatment. (**a**) Phospho-STAT3 (Y705, p-STAT3) protein expression in whole-BM cells. Densitometry ratios of p-STAT3 to total STAT3 were quantified. Whole-BM cells isolated from *Runx1*^+/+^ and *Runx1*^+/−^ mice were serum-starved for 1 h and subjected to 100 ng/ml G-CSF stimulation for the indicated duration. Mean±s.d. of the densitometry ratio and the representative western blot from three independent experiments is shown. (**b**) Graphical representation of the mean fluorescence intensity (MFI) of p-STAT3 within the KSL and KL compartments as assessed by Phosflow analysis. Sorted fractions were serum-starved for 1 h and subjected to 100 ng/ml G-CSF for 1 h. Data represent mean±s.d. (*n*=4/genotype). Results were pooled from two independent experiments. (**c**) Quantitative real-time PCR for *Csf3r* expression in KSL cells. Expression of *HPRT1* was used as endogenous control. Data represent mean±s.d. (*n*=3/genotype). (**d**) Graphical representation (left) and representative flow cytometry plots (right) of p-STAT3 in 32Dcl3 cells. RUNX1-GFP or GFP^*−*^ transfected 32Dcl3 cells were stimulated with 50 ng/ml G-CSF. The MFI of p-STAT3 and representative flow cytometry plots for GFP-positive transfected populations are shown. (**e**) Western blot analysis of p-STAT3 expression in HEK293T cells transfected with RUNX1 or control plasmid. Densitometry ratios of p-STAT3 to total STAT3 were quantified. Mean±s.d. of the densitometry ratio and the representative western blot from three independent experiments are shown. (**f**) Graphical representation of p-STAT3 in HEK293T cells. HEK293T cells were transfected with GFP empty vector or GFP-RUNX1 for at least 24 h before stimulation with 50 ng/ml oncostatin-M (OSM). The percentage of p-STAT3 positive cells (left) and MFI of p-STAT3 (right) were quantified. Data represent mean±s.d.. Three independent experiments were performed. Asterisks represent significant differences (*P<0.05, **P<0.01, ***P<0.001 and two-tailed Student's *t-*test).

G-CSF binds to its receptor and rapidly induces the phosphorylation of STAT3, which then translocates to the nucleus to induce gene transcription. We therefore assessed whether G-CSF hypersensitivity of *Runx1*^+/−^ mice was due to an increased phosphorylation of STAT3 at tyrosine 705 (p-STAT3) compared with *Runx1*^+/+^ mice. After 15 and 60 min of G-CSF treatment, p-STAT3 was significantly higher in whole-BM of *Runx1*^+/−^ mice than *Runx1*^+/+^ mice (Figure 3a). The magnitude of p-STAT3 activation among HSPCs and myeloid progenitor cells of *Runx1*^+/+^ and *Runx1*^+/−^ mice was also analyzed by comparing the mean fluorescence intensity by Phosflow analysis. Induction of p-STAT3 was also significantly higher in the KSL and KL compartments of *Runx1*^+/−^ mice than that of their *Runx1*^+/+^ counterparts (Figure 3b). Elevated STAT3 activation was not due to increased expression of *Csf3r* as the expression of *Csf3r* mRNA was indistinguishable in the *Runx1*^+/−^ and *Runx1*^+/+^ KSL fractions (Figure 3c).

To further support the notion that RUNX1 negatively regulate STAT3 activation, we tested the ability of RUNX1 to reduce G-CSF-induced STAT3 phosphorylation in 32Dcl3 myeloid cells. Overexpression of RUNX1 in 32Dcl3 cells resulted in a significant reduction in the mean fluorescence intensity of p-STAT3 (Figure 3d). Enforced expression of RUNX1 also reduced p-STAT3 induced by another cytokine, oncostatin-M (Figures 3e and f). Altogether, these results demonstrate that RUNX1 functions as a negative regulator of STAT3 signaling.

### RUNX1 haploinsufficiency results in reduced expression of *Pias3* and *Cxcr4*

**Figure 4:**
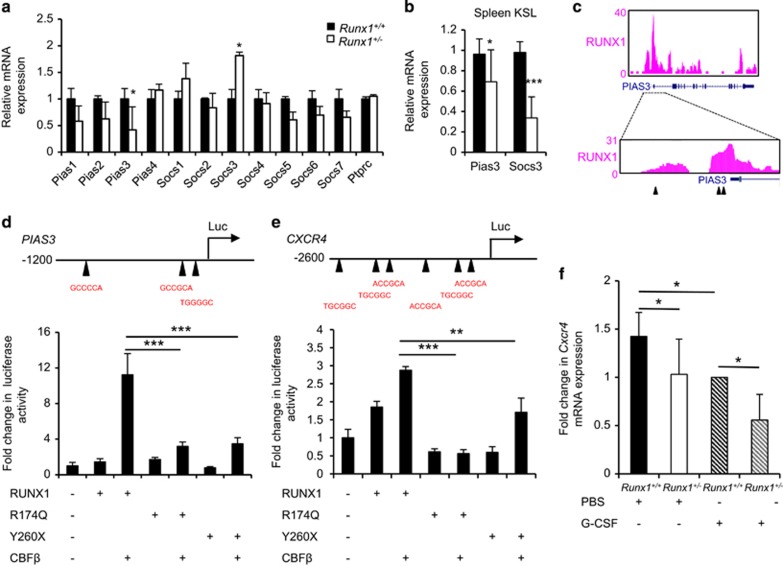
*Runx1* haploinsufficiency alters expression of key genes modulating cytokine response and hematopoietic stem/progenitor cells mobilization. (**a**) Expression profiles of *Pias*-, *Socs*-family genes and *Ptprc* within the KSL cells from the BM. Expression of *HPRT1* was used as endogenous control. (*n*=3/genotype). (**b**) Expression of Pias3 and Socs3 within the KSL cells from the spleen. Expression of *HPRT1* was used as endogenous control (*n*=5/genotype). (**c**) Occupancy of RUNX1 at *PIAS3* gene locus in human CD34+ cell-derived megakaryocytes. Black arrowheads represent RUNX1-binding sites. (**d** and **e**) Luciferase activity in HL-60 cells after co-transfection of either *PIAS3* (**d**) or *CXCR4* (**e**) reporter with the indicated *RUNX1*, *RUNX1*^*R174Q*^ (R174Q), *RUNX1*^*Y260X*^(Y260X) and *CBFB* plasmids. Black arrowheads on the luciferase constructs indicate the position of RUNX1-binding sites. Luciferase activity was normalized to Renilla internal control. Three independent experiments were performed. (**f**) Relative expression of *Cxcr4* mRNA in KSL cells. Mice were treated with 250 μg/kg/day G-CSF *in vivo* stimulation for three consecutive days. All data represent mean±s.d. *(Runx1*^+/+^mice phosphate-buffered saline (PBS) control, *n*=2; *Runx1*^+/−^ mice PBS control, *n*= 2; *Runx1*^+/+^mice G-CSF-treated, *n*= 3; *Runx1*^+/+^mice G-CSF-treated, *n*= 3). Asterisks represent significant differences (*P<0.05, **P<0.01 and two-tailed Student's *t*-test).

RUNX1 haploinsufficiency is likely to result in aberrant gene expression of RUNX1 target genes. The protein inhibitor of activated STAT (PIAS) and suppressor of cytokine signaling (SOCS) families of proteins are well-established negative regulators of STAT signaling. Therefore, we examined if these genes were differentially expressed in the KSL compartment of *Runx1*^+/+^ and *Runx1*^+/−^ mice. Expression of *Pias3*, a specific negative regulator of STAT3 was significantly reduced in the BM KSL cells of *Runx1*^+/−^ mice (Figure 4a). This reduced expression of *Pias3* was also observed in the spleen KSL cells from *Runx1*^+/−^ mice (Figure 4b). These results suggest that G-CSF hypersensitivity in *Runx1*^+/*−*^ HSPCs may be due to the reduced expression of *Pias3*. Surprisingly the expression of another feedback regulator, *Socs3*, was conversely upregulated in *Runx1*^+/−^ BM KSL cells; however, this upregulation of *Socs3* expression was not detected in the spleen KSL cells of *Runx1*^+/−^ mice (Figure 4b). It is known that secretion of G-CSF in the BM is much higher than that in the spleen.^25^ Therefore, our observation of *Socs3* upregulation in *Runx1*^+/−^ BM is probably an indication of active STAT3 signaling, but not a direct consequence of *Runx1*^+/*−*^ status.

The reduced expression of Pias3 in *Runx1*^+/−^ KSL cells prompted us to investigate if Runx1 is a direct regulator of Pias3 expression. Examination of publicly available chromatin immunoprecipitation-sequencing data set showed enrichment in the binding of RUNX1 at the promoter region of PIAS3 in both human CD34^+^ megakaryocytes^26^ (Figure 4c) and 12-*O*-tetradecanoylphorbol-13-actate-induced murine L8057 cells^27^ (data not shown). Motif search analysis revealed that 3 RUNX1-binding consensus sequences were found within close proximity in the human *PIAS3* promoter. To ascertain the role of RUNX1 in the regulation of PIAS3 expression, the −1.2-Kb promoter region covering these 3 RUNX1-binding sites were cloned upstream of a luciferase reporter. Transfection of RUNX1 and CBFβ strongly induced luciferase expression (Figure 4d). We tested two RUNX1 mutants, which were previously described in FPD/MM pedigrees,^3, 4^ R174Q and Y260X, for their ability to induce PIAS3 expression. Both R174Q and Y260X mutants were unable to transactivate luciferase (Figure 4d). Together, these results indicate that RUNX1 positively regulate the expression of PIAS3 in a DNA-binding dependent manner.

One of the most prominent phenotypes observed in *Runx1*^+/−^ mice after G-CSF stimulation was the enhanced mobilization of HSPCs. *CXCR4* is known to be a RUNX1 target gene and directly influences HSC retention in BM. Although RUNX1 transactivated the −2.6-KB promoter of human *CXCR4*, both mutants of RUNX1 (R174Q and Y260X) were unable to do so (Figure 4e). *Cxcr4* expression was marginally reduced at the mRNA level when comparing phosphate-buffered saline-treated *Runx1*^+/−^ KSL cells to *Runx1*^+/+^ KSL cells (Figure 4f). Treatment with G-CSF resulted in a further reduction of the mRNA expression of Cxcr4 in *Runx1*^+/−^ KSL cells (Figure 4f). This G-CSF-mediated suppression of Cxcr4 expression may serve to augment the egress of KSL cells from the BM in *Runx1*^+/−^ mice.

### RUNX1 attenuates STAT3 signaling by physical interaction

**Figure 5:**
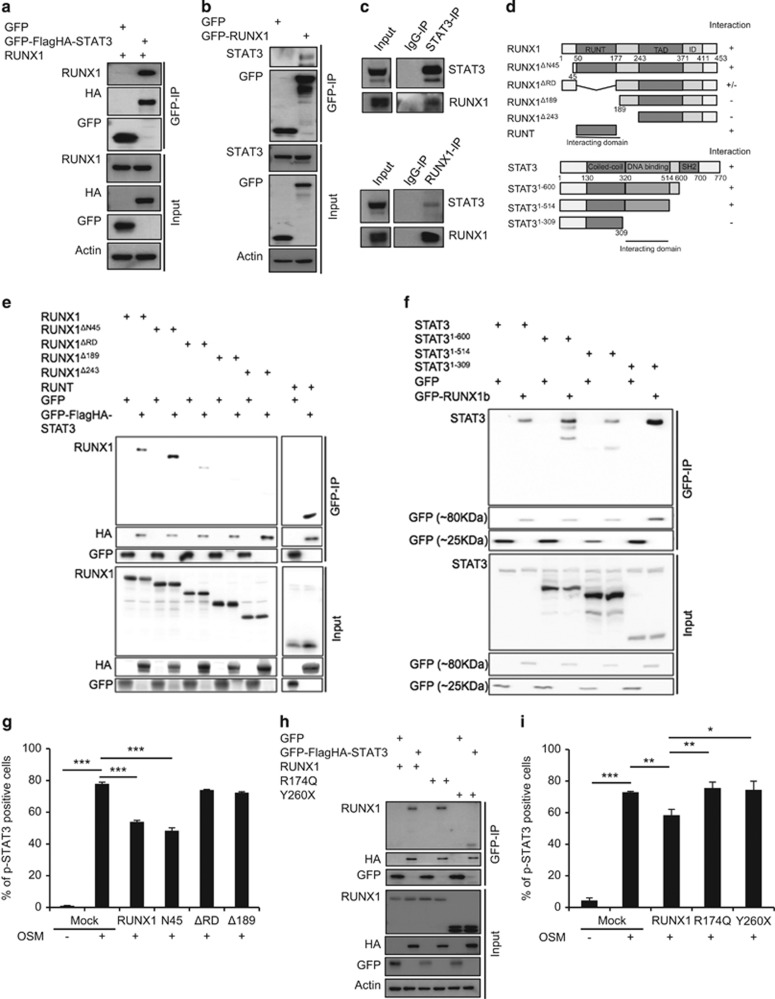
RUNX1 physically interacts with STAT3 to attenuate phosphorylation of STAT3 after cytokine treatment. (**a**) Co-immunoprecipitation of RUNX1 with STAT3 in HEK293T cells. GFP-tagged FLAGHA-STAT3, RUNX1, or GFP-expressing plasmids were overexpressed in HEK293T. Lysates were co-immunoprecipitated using GFP beads, and western blots were probed with indicated antibodies. (**b**) Co-immunoprecipitation of endogenous STAT3 with RUNX1 in HEK293T cells. EGFP-tagged RUNX1, or EGFP-expressing plasmids were overexpressed in HEK293T by lipofection. Lysates were co-immunoprecipitated using GFP beads, and western blots were probed with indicated antibodies. (**c**) Co-immunoprecipitation of RUNX1 with STAT3 in Jurkat cells. Lysates were co-immunoprecipitated with the indicated antibodies. Western blots were probed with the indicated antibodies. Representative blots from three independent experiments are shown. (**d**) Schematic diagram of mutant constructs of RUNX1 and STAT3. (**e** and **f**) Co-immunoprecipitation of RUNX1 mutant constructs with STAT3 (**e**), and reciprocal co-immunoprecipitation of STAT3 mutant constructs with RUNX1 (**f**). Lysates were co-immunoprecipitated using GFP beads, and western blots were probed with indicated antibodies. (**g**) Attenuation of p-STAT3 by RUNX1 mutants. HEK293T were transfected with indicated RUNX1 constructs for 24 h and treated with 50 ng/ml oncostatin-M (OSM) for 30 min. The percentage of p-STAT3-positive cells was determined by Phosflow analysis. Mean±s.d. of three independent experiments is shown. (**h**) Interaction of FPD/MM patients-associated RUNX1 mutants with STAT3. Lysates were co-immunoprecipitated using GFP beads, and western blots were probed with indicated antibodies. (**i**) Attenuation of p-STAT3 by RUNX1 or FPD/MM patients-associated RUNX1 mutants. HEK293T were transfected with RUNX1, R174Q or Y260X for 24 h and treated with 50 ng/ml OSM for 30 min. The percentage of p-STAT3-positive cells was determined by flow cytometry. Mean±s.d. of three independent experiments is shown. Asterisks represent significant differences (*P<0.05, **P <0.01, ***P<0.001 and two-tailed Student's *t*-test).

Several groups have reported that STAT family proteins interact with RUNX proteins to attenuate their transcriptional function.^28, 29^ As such, we evaluated if RUNX1 is able to modulate STAT3 activation via physical interaction. The physical interaction between RUNX1 and STAT3 was tested using GFP-pulldown assay. RUNX1 was co-immunoprecipitated with exogenous GFP-tagged STAT3 (Figure 5a). Similarly, STAT3 was co-immunoprecipitated together with exogenous GFP-tagged RUNX1 (Figure 5b). Endogenous interaction between RUNX1 and STAT3 was also confirmed in Jurkat cells (Figure 5c). These results demonstrate that RUNX1 physically interacts with STAT3.

We further used various RUNX1 and STAT3 deletion constructs to determine the interacting domains of RUNX1 and STAT3 (Figure 5d). Unlike full-length RUNX1, RUNX1 with deleted Runt domain (RUNX1^ΔRD^), RUNX1^Δ189^ and RUNX1^Δ243^ failed to interact with STAT3, whereas Runt domain alone was successfully co-immunoprecipitated with STAT3 (Figure 5e). Therefore, RUNX1 interacts with STAT3 predominantly via the Runt domain. In a reciprocal experiment, STAT3/RUNX1 interaction was preserved in deletion mutants STAT3^1-514^ and STAT3^1-600^, but disrupted in the STAT3^1-309^ mutant (Figure 5f). Hence the DNA-binding domain of STAT3 is likely to be essential for the interaction with RUNX1. We also tested if the various RUNX1 mutants have the ability to attenuate p-STAT3. Only full-length RUNX1 and RUNX1^ΔN45^ mutant retained the ability to attenuate the phosphorylation of STAT3, whereas all non-interacting mutants failed to do so (Figure 5g).

We next tested the ability of FPD-related RUNX1 point mutants to associate with STAT3 using similar co-immunoprecipitation experiments. Both R174Q and Y260X mutants were able to interact with exogenous STAT3 (Figure 5h). However, both R174Q and Y260X were unable to attenuate STAT3 phosphorylation after treatment with oncostatin-M (Figure 5i). Our data suggest that although the Runt domain is sufficient for STAT3 interaction, both the Runt domain and carboxyl terminus of RUNX1 are required to exert negative regulation on STAT3.

### FPD patient with *RUNX1* point mutation displays G-CSF hypersensitivity

**Figure 6:**
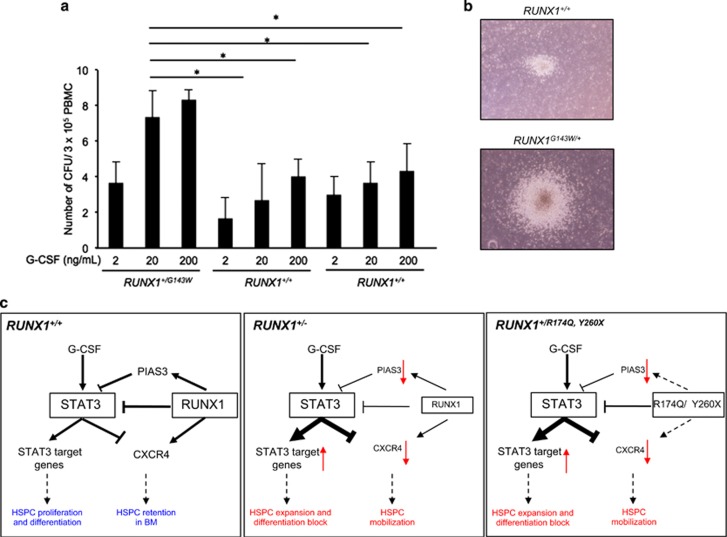
Peripheral mononuclear cells from FPD patient show G-CSF hypersensitivity *in vitro*. (**a** and **b**) Colony-forming potential of PB mononuclear cells (PBMC) in the presence of increasing amount of G-CSF. Three hundred thousand PBMCs derived from two healthy donors and FPD patient harboring point mutation in Runt domain (*RUNX1*^*+/G143W*^) were treated with indicated concentrations of G-CSF. Mean±s.d. is shown. In **b**, the difference in size of colony was shown. (**c**) Schematic diagrams depicting how RUNX1 negatively regulate STAT3 signaling (left). *RUNX1* haploinsufficiency (center) and point mutation (right) results in reduced *CXCR4* and *PIAS3* transcription and physical sequestration of STAT3 by RUNX1. Asterisks represent significant differences (*P<0.05 and two-tailed Student's *t*-test).

Finally, we sought to test whether FPD patients harboring *RUNX1* point mutations present similar G-CSF hypersensitivity like the *RUNX1*^+/−^ mice. PB mononuclear cells derived from a FPD patient with a *RUNX1*^*G143W*^ mutation were able to form two- to threefold more colonies than healthy volunteers with wild-type *RUNX1* in increasing doses of G-CSF (Figure 6a). In addition, morphological analysis of the colonies revealed that the colonies from the FPD patient were larger in size compared with healthy volunteers, suggesting that the proliferation rate of these RUNX1 mutant cells were increased (Figure 6b). Consistent with the observations in *Runx1*^+/−^ mice, *RUNX1* haploinsufficiency in the FPD patient also resulted in similar G-CSF hypersensitivity.

## Discussion

In this study, we show that *Runx1* haploinsufficiency causes G-CSF hypersensitivity that manifests in two major phenotypes: (1) expansion and mobilization of HSPCs and (2) differentiation block in G-CSF-induced granulopoiesis. In response to G-CSF, *Runx1*^+/−^ KSL cells sustained greater activation of STAT3 than Runx1^+/+^ KSL cells. Our data depicts a model whereby RUNX1 exerts a negative regulation on STAT3 signaling in both transcriptional and non-transcriptional manners (Figure 6c, left). Our model shows that RUNX1 transcriptionally regulates PIAS3 and physically interacts with STAT3 to inhibit its function. The combined influence of these regulations ensures proper control of gene expression for HSPC proliferation, migration and differentiation. *RUNX1* alterations in FPD patients, such as *RUNX1*^+/*−*^ (Figure 6c, middle) or point mutants (Figure 6c, right), may result in weakened suppression of STAT3 signaling, and consequently aberrant HSPC expansion and delayed myeloid differentiation.

Recently, the Encyclopedia of DNA Elements (ENCODE) project reported a significant overrepresentation of the enrichment of RUNX1/STAT1/STAT3-binding motifs within the regulatory elements.^30^ Coexistence of RUNX1− and STAT3-binding motifs in close proximity within the regulatory elements of various genes were reported to permit positive cooperative effects in the control of gene expression.^31, 32^ Such synergism between RUNX1 and STAT3 appears to be highly relevant in the context of several epithelial carcinomas.^33, 34^ Scheitz *et al.*^34^ demonstrated that RUNX1 promotes STAT3 signaling via suppression of SOCS3 and SOCS4 expression. The loss of RUNX1 resulted in a reduction of STAT3 signaling and prevented the initiation and maintenance of epithelial tumors. In contrast, RUNX and STAT family proteins were shown to physically interact and mutually inhibit their own transcriptional function.^28, 29, 35^ Although our data show that RUNX1 suppresses STAT3 signaling induced by G-CSF in the context of HSPCs, we do not deny the possibility that synergism between RUXN1 and STAT3 is also important. The net-effect of positive and negative regulations by RUNX1 will influence the eventual activation of G-CSF-induced gene expression programs.

We report that *Runx1*^+/−^ mice showed G-CSF hypersensitivity due to insufficient sequestration of STAT3 by RUNX1. Importantly, an intact Runt domain is essential for RUNX1 to inhibit the transcriptional function of STAT3. In previous studies, both Stat1 and Stat3 were reported to interact and sequester Runx2 in the cytoplasm.^29, 35^ Stat5 was further shown to interact and attenuate the function of all members of RUNX transcription factors.^28^ Taken together, all members of RUNX and STAT family transcription factors interact and mutually moderate the transcriptional activity of each other. The Runt domain is highly conserved across the different RUNX family members; thus RUNX2 and RUNX3 are likely to use Runt domain to interact with STAT family proteins. *Runx3* conditional knockout mice also showed similar G-CSF hypersensitivity.^22^ Therefore it strongly suggests that RUNX family proteins function as negative regulators of STAT signaling by physical interaction.

*RUNX1* haploinsufficiency may result in exaggerated cytokine responses during infection, a short period of time when endogenous level of G-CSF and other cytokines can be greatly elevated for emergency granulopoiesis.^36^ RUNX1 haploinsufficient individuals may experience minor granulocyte differentiation blockage and accumulate HSPCs in the spleen and PB over an extended period of time. Of the many progenitor populations, the most prominently expanded population in *Runx1*^+/−^ mice, namely GMPs, have been regarded as candidate targets for oncogenic transformation in leukemia.^37^ Furthermore, GMPs were efficiently transformed into leukemia-initiating cells by retroviral transduction of MLL-AF9.^38^ Therefore, the sustained expansion of preleukemic GMPs in FPD patients may greatly elevate the likelihood of acquiring cooperating mutations to develop full-blown leukemia. As FPD/MM patients exhibit clinical heterogeneity in both the severity of platelet defects and age of leukemia onset,^39^ extensive variability in the age of leukemia onset can be explained by the time at which the cooperating mutation was acquired and the severity of these cooperative hits. Mutations in RUNX1 are also considered to be initiating event for onset of various sporadic hematological malignancies such as myelodysplastic syndrome or chronic myelomonocytic leukemia. We speculate that G-CSF-induced preleukemic GMPs expansion may also contribute to the leukemogenesis of these diseases.

FPD has been reported to be under-diagnosed due to the large variation in the severity of platelet defects. *RUNX1* mutation carriers who do not display distinct clinical features or family history are typically not screened for *RUNX1* mutations. This under-diagnosis has repeatedly led to dire consequences: affected siblings with little or no detectable platelet abnormalities acted as donors for HSCT and the recipients eventually developed leukemia due to donor-derived *RUNX1* mutation.^39^ Furthermore, mutations in the *RUNX1* coding regions are not the only scenario whereby *RUNX1* haploinsufficiency can occur. A significant number of FPD pedigrees did not show mutation in the *RUNX1* exons, implying that *cis*-regulatory elements may also be able to reduce RUNX1 expression.^40^ Establishment of new accurate diagnosis methods is warranted. Our study shows that G-CSF hypersensitivity is observed *in vitro* irrespective of the types of *RUNX1* alterations. As granulocyte-macrophage CSF hypersensitivity is also used as a criterion to diagnose juvenile myelomonocytic leukemia,^41^ we propose that G-CSF hypersensitivity would serve as a complementary test to detect affected FPD subjects, including cryptic cases.
