# Supplementary material for: RUNX1 haploinsufficiency results in granulocyte colony-stimulating factor hypersensitivity
Source: Blood Cancer J. 2016 Jan 8;6(1):e379–. doi: 10.1038/bcj.2015.105 (PMC4742622; doi:10.1038/bcj.2015.105)
Supplement: Supplementary Information [file bcj2015105x1.docx]

**Supplemental methods**

**Phosflow analysis**

The cKit^+^Sca-1^+^Lin^-^ (KSL, HSPC) and cKit^+^Sca-1^-^Lin^-^ (KL, myeloid progenitors) compartments were sorted using FACSAria and subjected to serum starvation for 1 hour at 37^o^C. The cells were then incubated with 100 ng/mL mG-CSF in α-MEM for 30 minutes. Transfection of 32Dcl3 cells with RUNX1-GFP plasmid was performed using Neon Transfection system (Life Technologies). After 48 hours, the cells were treated with 50 ng/mL G-CSF for 60 minutes. Alternatively, RUNX1-transfected HEK293T cells were treated with 50 ng/mL human oncostatin-M (Peprotech) for 60 minutes. Cells were then fixed in 2% paraformaldehyde-PBS (Sigma-Aldrich) for 10 minutes at room temperature. After washing twice with PBS, ice-cold methanol was added drop-wise to the cell pellet under constant agitation and stored in -20^o^C overnight. Cells were washed in PBS and resuspended in 1% bovine serum albumin-PBS. The cells were stained with p-STAT3 (Y705) antibody (BD Biosciences, #560312) according to the recommended instructions.

**Co-immunoprecipitation assay**

HEK293T cells were transfected with various RUNX1 and STAT3 constructs using lipofectamine 2000 (Life Technologies) for 24 to 48 hours. Cells were washed twice with ice-cold PBS and then lysed with Co-IP buffer (10 mM Tris-Cl pH 7.5, 150 mM NaCl, 0.5 mM EDTA, 0.5% NP-40) supplemented with 50 U/mL Benonase nuclease (Novagen), 1 mM phenylmethylsulfonyl fluoride (PMSF), 1X protease inhibitor cocktail (Nakalai Tesque Inc.) and HALT phosphatase inhibitor (Pierce). Protein lysates were sonicated and debris was removed by centrifugation. Protein concentrations were normalized and incubated with GFP-trap beads according to manufacturer’s protocol (Chromotek). For the endogenous IP, 1 mg of Jurkat cell protein lysate was incubated with 1 μg of anti-STAT3 (Santa Cruz biotechnology, #Sc-482), anti-RUNX1 (Abcam, #Ab35962) or control IgG antibody. Protein A/G agarose beads (Calbiochem, #IP-10) were added into the lysates and incubated for 3 hours. Beads were washed 5 times with Co-IP buffer. Bound proteins were eluted with 2X SDS sample buffer and then subjected to western blot analysis.

**Western blot analysis**

Cells were lysed in 5x packed volume of 4% SDS solution and protein concentration was estimated using BCA protein assay reagent (Pierce). Equal amounts of protein were loaded and resolved using 8% to 12% SDS-PAGE gels. The membrane was blocked using PBS with 2% BSA (Sigma-Aldrich) and 0.1 % Tween-20 (Sigma-Aldrich). Antibodies against RUNX1 (MBL, #D208-3, 1:1000; Active Motif, #39000, 1:1000), GFP (Abcam, #290-50, 1:4000), HA (Santa Cruz biotechnology, #sc-7392, 1:1000), β-Actin (Sigma, A1978, 1:5000), STAT3 (Cell Signaling Technology, #12640S, 1:1000; Santa Cruz biotechnology, #Sc-482, 1:1000) and phospho-STAT3 (Tyr705) (Cell Signaling Technology, #4113S, 1:1000) were used.

**Supplemental table 1**

List of antibodies used for flow cytometry.

| Antigen | Antibody Clone |
| --- | --- |
| CD3 | 145-2C11 |
| CD4 | H129.19 |
| CD8 | 53.6.7 |
| B220 | RA3-6B2 |
| Gr-1 | RB6.8C5 |
| Mac-1 | M1/70 |
| Ter-119 | TER-119 |
| CD127 | A7R34 |
| CD71 | C2 |
| CD41 | MWReg30 |
| CD61 | 2C9.G2 |
| CD16/32 | 2.4G2 |
| c-Kit | 2B8 |
| Sca-1 | D7 |
| Flt-3 | A2F10 |
| CD45 | 30-F11 |
| CD34 | RAM34 |
| STAT3 (pY705) | 4/P-STAT3 |

**Supplemental table 2**

List of primers and probes used for real-time quantitative PCR.

| Gene | Dye |  | Sequence (5' -3') |
| --- | --- | --- | --- |
| *Pias1* | SYBR | Forward | AGTGCGGAACTAAAGCAAATGG |
|  |  | Reverse | TTTTCTGAGGGAACCGCCTC |
| *Pias2* | SYBR | Forward | TGCCCAGTGTGACTTCAGTT |
|  |  | Reverse | GAGGTGAGACTGGGGATCAA |
| *Pias3* | SYBR | Forward | CCAGGCCAGAGCTTCATGGATAC |
|  |  | Reverse | TGCCTTTCCCCAAGAGGCT |
| *Pias4* | SYBR | Forward | GAGAAGCTTCGCCTAGACCC |
|  |  | Reverse | CGACAGCAGCCCATCTATGA |
| *Socs1* | SYBR | Forward | GGCTCACTGCCTCTGTCTC |
|  |  | Reverse | AAGGTGCGGAAGTGAGTGTC |
| *Socs2* | SYBR | Forward | CCCGTGTGAAATCCAAGGCT |
|  |  | Reverse | AGGAGTGCAAGGGCAATGTC |
| *Socs3* | SYBR | Forward | TTGAGCGTCAAGACCCAGTC |
|  |  | Reverse | CGTGGGTGGCAAAGAAAAGG |
| *Socs4* | SYBR | Forward | CCACACCCAGATCGACTACG |
|  |  | Reverse | TGGCTTTCCTTCCAGCAGAG |
| *Socs5* | SYBR | Forward | TGAACCCCAACAGATGTCCG |
|  |  | Reverse | CACAGTTTTGGTTCCGCCTG |
| *Socs6* | SYBR | Forward | CCTTCAGTACACCGTGCCTT |
|  |  | Reverse | GGCTCTGCAACATGACTCCT |
| *Socs7* | SYBR | Forward | TATCAGTGGGACGCTGCCTA |
|  |  | Reverse | CTGCAAAGCTGCTTGAGTCG |
| *Hprt1* | SYBR | Forward | CAGTCCCAGCGTCGTGATTAG |
|  |  | Reverse | AAACACTTTTTCCAAATCCTCGG |
| *Csf3r* | SYBR | Forward | CCTGGATGATAGAACCTAACGGG |
|  |  | Reverse | CTCTCCAGCGAAGGTGTAGACA |
| *PIAS3* | SYBR | Forward | GATTGGGAAGGGCACAGG |
|  |  | Reverse | ACTTCCCCTGCCTCCTACTCC |
| *HPRT1* | SYBR | Forward | ATTCTTTGCTGAGCTGGATT |
|  |  | Reverse | CCAATTACTTTTATGTCCCCTGTTG |
| *Cxcr4* | Taqman | ID | Mn99999051_m1 |
| *Ptprc* | Taqman | ID | Mn01293577_m1 |
| *Gapdh* | Taqman | ID | Mn99999915_m1 |

**Supplemental figure legends**

**Supplemental figure 1. *Runx1^+/-^* mice show mild defects in platelet production.**

(**A**) Complete blood count (CBC) performed on 6 to 8 week old *Runx1^+/+^* (n= 11) and *Runx1^+/-^* (n= 14) mice. Mean ± SD of leukocyte count, hemoglobin and platelet quantities are shown.

(**B, C**) Frequencies of cells in the bone marrow (BM) (**B**) and spleen (**C**) of 6 to 8 week old *Runx1^+/+^* and *Runx1^+/-^* mice. B cells, B220^+^CD19^+^; Myeloid, Mac-1^+^ and Gr-1^+^; Megakaryocyte (MgK), CD41^+^CD61^+^; T cells, CD3^+^. Mean ± SD are shown (n= 7/genotype).

(**D, E**) Representative flow cytometry plots (left) and graphical representation (right) of the frequencies HSPC compartments gated from viable Lineage^-^ cells of *Runx1^+/+^* and *Runx1^+/-^* mice in the BM (**D**) and spleen (**E**) at 6 to 8 week old. All data represent mean ± SD (n= 7/genotype).

Asterisks represent significant differences (* *p* < 0.05, ** *p* < 0.01, *** *p* < 0.001, 2-tailed Student’s t test).

**Supplemental figure 2. *Runx1^+/-^* mice exhibit acute granulocyte colony-stimulating factor hypersensitivity *in vivo*, related to Figure 2.**

(A- C) Representative flow cytometric plots of the HSPC compartments in the bone marrow (BM) 24 hours after 250 μg/kg/day granulocyte colony-stimulating factor (G-CSF) stimulation in vivo for 3 consecutive days. Plots of 0.2 – 1 x 10^6^ cells were gated from Lineage^-^ cells (A), c-Kit^+^Sca-1^+^Lineage^-^ (KSL) cells (B) and, c-Kit^+^Sca-1^-^Lineage^-^ (KL) cells (C).

(D, E) Graphical representation of the frequencies of various HSPC compartments in the BM, 24 hours after 250 μg/kg/day G-CSF stimulation in vivo for 3 consecutive days. All data represent mean ± SD (Runx1^+/+^ mice PBS control, n= 4; Runx1^+/-^ mice PBS control, n= 4; Runx1^+/+^ mice G-CSF-treated, n= 6; Runx1^+/+^ mice G-CSF-treated, n= 6). Abbreviations: HSPC, hematopoietic stem and progenitor cell (c-Kit^+^Sca- 1^+^Lineage^-^); LT-HSC, long-term hematopoietic stem cell (c-Kit^+^Sca- 1^+^Lineage^-^CD34^-^Flt3^-^); ST-HSC, short-term hematopoietic stem cells (c-Kit^+^Sca-1^+^Lineage^-^CD34^+^Flt3^-^); MPP, multipotent progenitor (c-Kit^+^Sca-1^+^Lineage^-^CD34^+^Flt3^+^); CMP, common myeloid progenitor (c-Kit^+^Sca-1^-^Lineage^-^CD34^+^FcγR^lo^); GMP, granulocyte macrophage progenitor (c-Kit^+^Sca-1^-^Lineage^-^CD34^+^FcγR^hi^); MEP, megakaryocyte erythroid progenitor (c-Kit^+^Sca-1^-^Lineage^-^CD34^-^FcγR^lo^).

Asterisks represent significant differences (*, p < 0.05, Student’s t test).

**Supplemental figure 3. *Runx1^+/-^* mice sustain hematopoietic stem and progenitor mobilization at 72 hours post granulocyte colony-stimulating factor stimulation, related to Figure 2.**

(A- C) Graphical representation of the frequencies of HSPC compartments in the bone marrow (BM) gated from Lineage^-^ cells (A), LT-HSC, ST-HSC and MPP compartments gated from KSL cells (B), and CMP, GMP and MEP compartments gated from KL cells (C) 72 hours after *in vivo* granulocyte colony-stimulating factor (G-CSF) stimulation (250 μg/kg/day for 3 days).

(D, E) Graphical representation of the frequencies of HSPC compartments (D) and GMP (E) in the spleen 72 hours after *in vivo* G-CSF stimulation (250 μg/kg/day for 3 days). All data represent mean ± SD (*Runx1^+/+^* mice G-CSF-treated, n= 4; *Runx1^+/-^* mice G-CSF-treated, n= 4).

Abbreviations see supplemental figure 2 legends. Asterisks represent significant differences (* *p* < 0.05, 2-tailed Student’s t test).
