# Supplementary figures and images for: RUNX1 haploinsufficiency results in granulocyte colony-stimulating factor hypersensitivity
Source: Blood Cancer J. 2016 Jan 8;6(1):e379–. doi: 10.1038/bcj.2015.105 (PMC4742622; doi:10.1038/bcj.2015.105)

Supplemental Figure 1

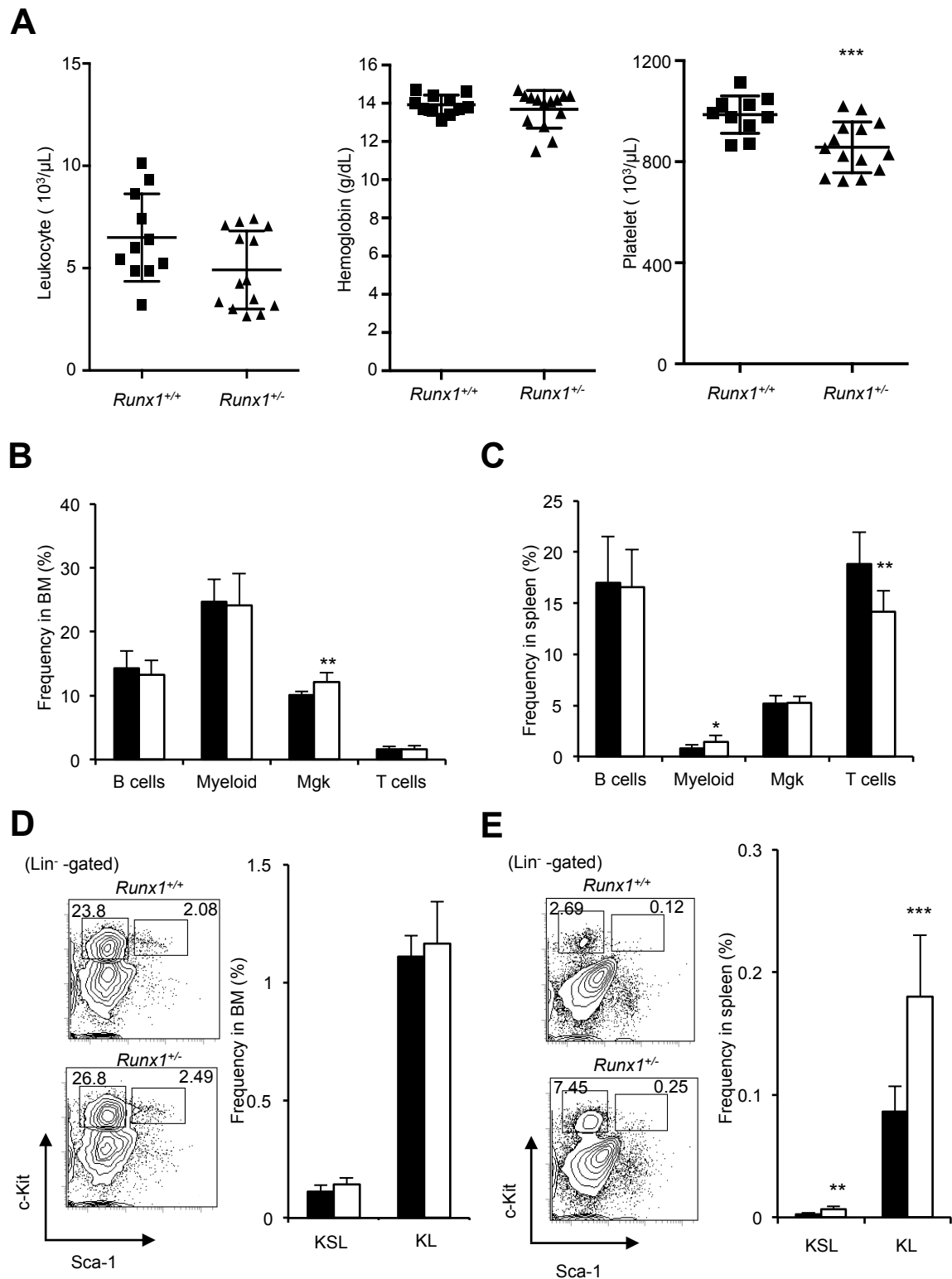

Supplemental Figure 2

A

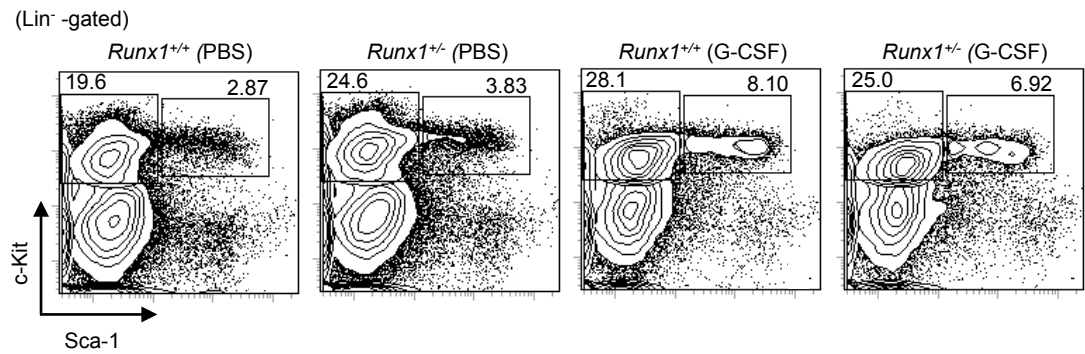

B

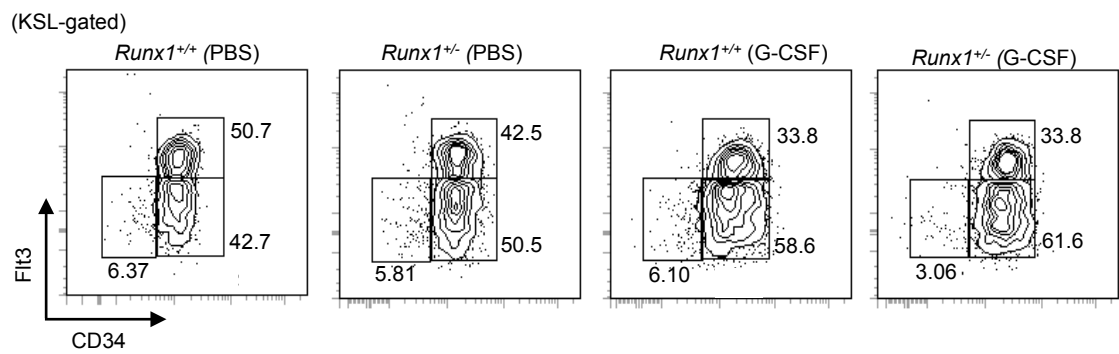

C

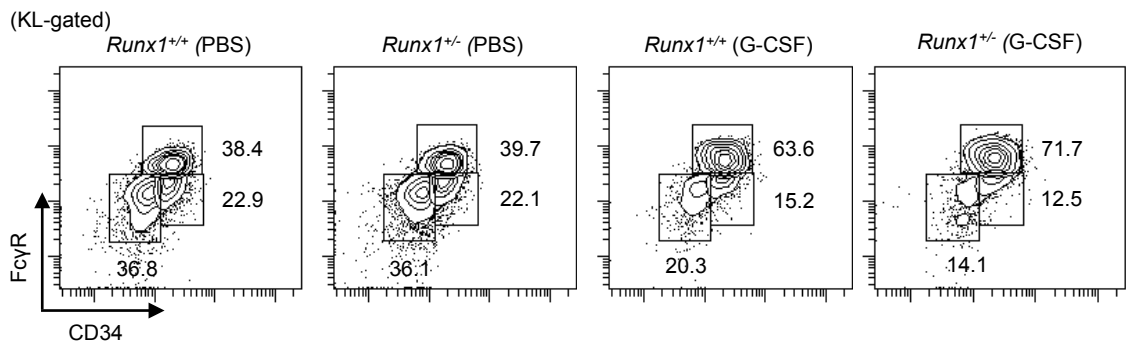

D

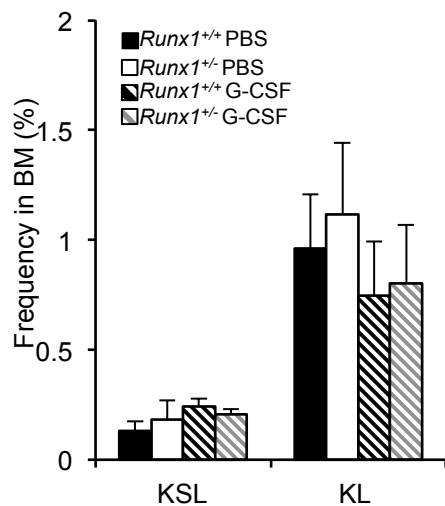

E

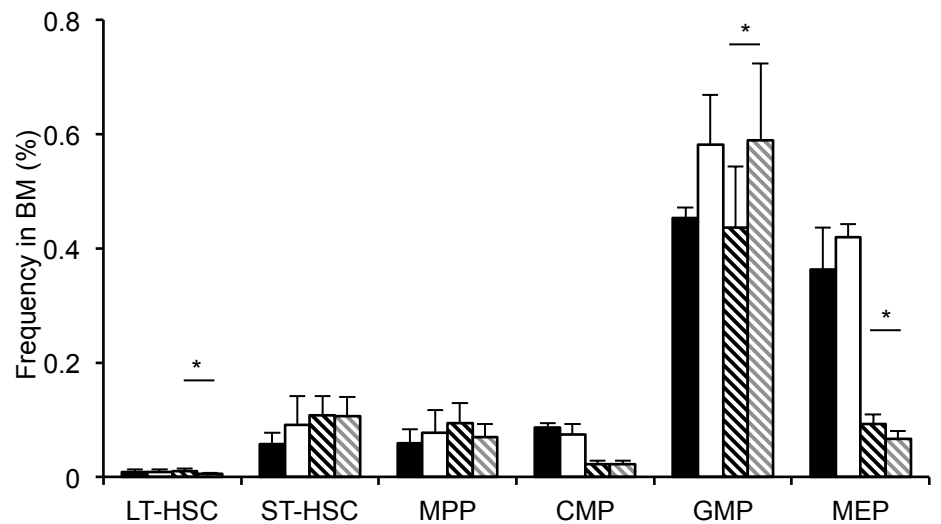

Supplemental Figure 3

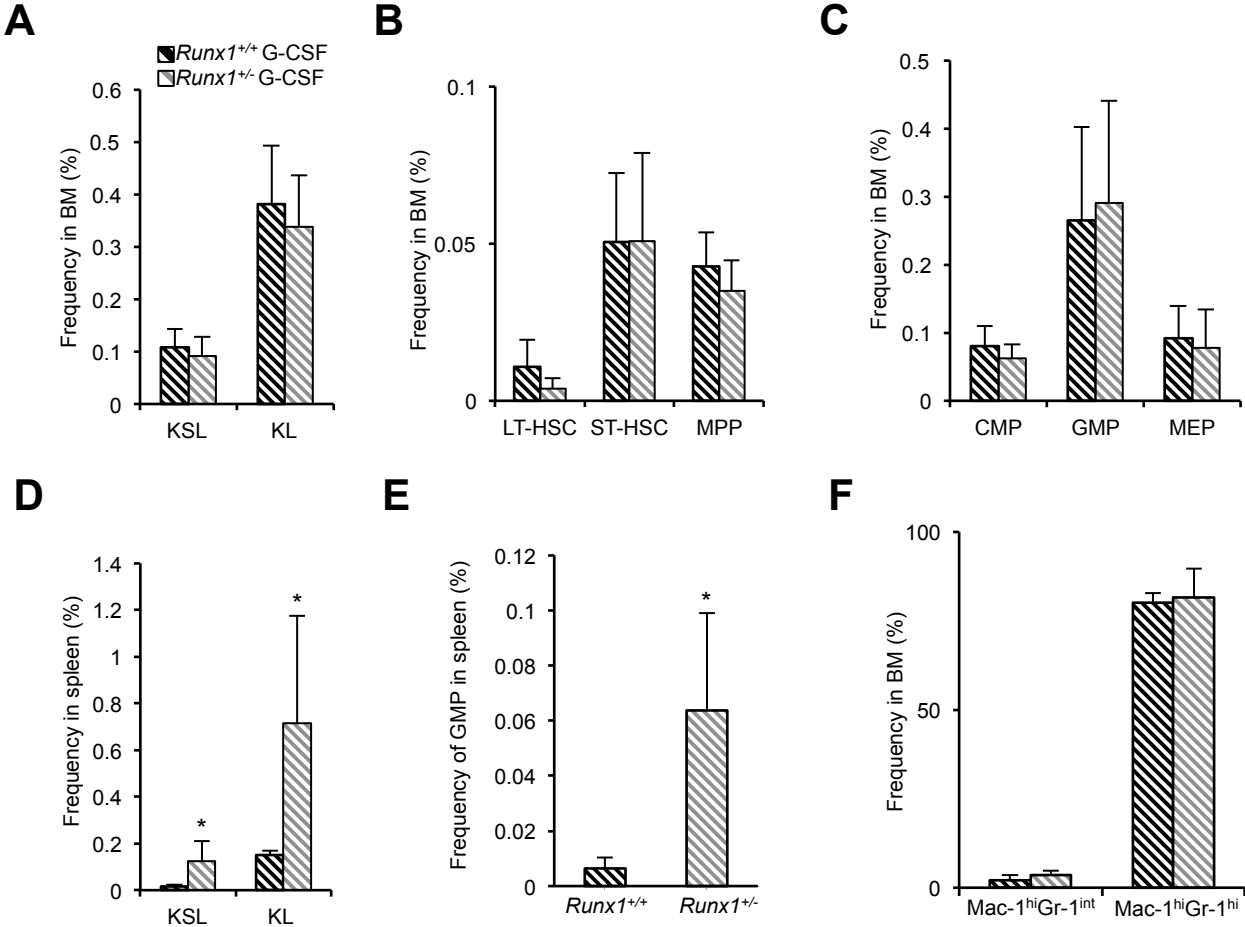

Supplement: Supplementary Figures 1-3 [file bcj2015105x2.pdf]
